# Development of enhanced ethanol ablation as an alternative to surgery in treatment of superficial solid tumors

**DOI:** 10.1038/s41598-017-09371-2

**Published:** 2017-08-18

**Authors:** Robert Morhard, Corrine Nief, Carlos Barrero Castedo, Fangyao Hu, Megan Madonna, Jenna L. Mueller, Mark W. Dewhirst, David F. Katz, Nirmala Ramanujam

**Affiliations:** 1 Department of Biomedical Engineering, Duke University, Durham, North Carolina United States of America; 2 Department of Radiation Oncology, Duke University, Durham, North Carolina United States of America; 3 Department of Obstetrics and Gynecology, Duke University, Durham, North Carolina United States of America; 4 Department of Pharmacology and Cancer Biology, Duke University, Durham, North Carolina United States of America; 5 Duke Global Health Institute, Duke University, Durham, North Carolina United States of America

## Abstract

While surgery is at the foundation of cancer treatment, its access is limited in low-income countries. Here, we describe development of a low-cost alternative therapy based on intratumoral ethanol injection suitable for resource-limited settings. Although ethanol-based tumor ablation is successful in treating hepatocellular carcinomas, the necessity for multiple treatments, injection of large fluid volumes, and decreased efficacy in treatment of non-capsulated tumors limit its applicability. To address these limitations, we investigated an enhanced ethanol ablation strategy to retain ethanol within the tumor through the addition of ethyl cellulose. This increases the viscosity of injected ethanol and forms an ethanol-based gel-phase upon exposure to the aqueous tumor environment. This technique was first optimized to maximize distribution volume, using tissue-simulating phantoms. Then, chemically-induced epithelial tumors in the hamster cheek pouch were treated. As controls, pure ethanol injections of either four times or one-fourth the tumor volume induced complete regression of 33% and 0% of tumors, respectively. In contrast, ethyl cellulose-ethanol injections of one-fourth the tumor volume induced complete regression in 100% of tumors. These results contribute to proof-of-concept for enhanced ethanol ablation as a novel and effective alternative to surgery for tumor treatment, with relevance to resource-limited settings.

## Introduction

In 2012 cancer caused over 5 million deaths, including 10% of all deaths before the age of 75 in developing countries^1^. While often treatable in developed countries, cancer remains a lethal diagnosis in many developing countries, as evidenced by the high ratio of mortality-to-incidence rates: 0.48 in developed countries, vs. 0.66 in developing countries^1^. This disparity widens in comparison of, for example, the United States and sub-Saharan Africa (0.35 vs. 0.73, respectively)^2^. Discrepancies in mortality-to-incidence rates are generally attributed to lack of access to basic cancer treatment resources in developing countries^3^.

The limited access to surgery in developing countries is due to shortages in both equipment and personnel^4^. A survey of 132 district-level facilities in eight low- and middle-income countries found that only 32% reported consistent availability of anesthesia machines, and only 36% had constant access to electricity^5^. Such shortages are further exacerbated by lack of trained medical personnel; a large majority of sub-Saharan countries do not meet the World Health Organization’s recommendations for numbers of doctors and nurses per capita^6^. Because of these obstacles, nine out of ten people in developing countries do not have access to basic surgical care^7^.

Tumor ablation, involving direct destruction of tumor cells, has potential to be an effective alternative to surgery for healthcare systems in developing countries. The ideal therapy for low-resource settings should be highly effective at treating localized lesions, portable, ultra-low-cost, and not depend on regular access to electricity. While microwave ablation^8^ and radiofrequency ablation^9^ are often effective tumor treatments, both require regular access to electricity and specialized equipment. So-called “cold coagulation,” which uses a heated probe to destroy malignant tissue (here, the tissue remains much cooler than it becomes after radiofrequency or microwave ablation) is relatively inexpensive and portable^10^. However, it treats only superficial lesions (up to a depth of 3.5 mm), and it requires access to electricity^11^. While battery-powered hand-held thermo-coagulators have been developed, these devices cost approximately $500–$1000 and require a consistent supply of batteries^12^. Cryotherapy, which induces necrosis by freezing malignant tissue, does not require access to electricity, but requires specialized equipment and potentially hard-to-supply compressed gas tanks and is not portable^13, 14^. Further, it only treats superficial lesions (to an average depth of 3.4 mm)^15^.

An alternative therapy, that meets many of the aforementioned constraints, is ethanol ablation. This employs direct injection of ethanol into malignant tissue to induce necrosis through protein denaturation and cytoplasmic dehydration^16^. Ethanol ablation is currently used to treat hepatocellular carcinomas, and yields 5-year survival rates comparable to surgical resection^17^. While most commonly applied in the liver, ethanol ablation has also been successfully employed in treatment of cardiomyopathies^18^, parathyroid^19^ and pancreatic^20^ tumors, adrenal metastases^21^, and metastatic pelvic lymph nodes^22^. Ethanol ablation is especially appealing for use in developing countries because it can be locally available and ultra-low-cost (<$5 per treatments), requires no specialized equipment, is highly portable, and can effectively treat relatively large lesions up to 5 cm in diameter^23–26^.

A shortcoming of ethanol ablation, however, is its reduced efficacy in treatment of non-capsulated tumors (i.e., tumors which are not surrounded by a fibrous capsule)^23^. This diminished efficacy could derive from increased crack formation^27^, which allows injected ethanol to escape the tumor, and reduces accumulation near the injection site. Additionally, effectiveness of contemporary tumor ablation often requires multiple treatment sessions and injection of large volumes of ethanol^28^ which risks damage to surrounding tissue. These drawbacks limit its translatability to low-resource settings.

Given the potential utility but known drawbacks of ethanol as an ablative agent, the goals of this study were to create a strategy to retain ethanol within malignant tissue, focusing upon maximizing the efficacy of single-dose tumor treatment. These were achieved by adding ethyl cellulose to injected ethanol. Ethyl cellulose is an ethanol-soluble and water-insoluble cellulose-derivative. When added to ethanol, it increases viscosity of the mixture, which has been shown to increase intratumoral injection efficacy^29^. Upon introduction to the aqueous tumor environment, the ethyl cellulose-ethanol mixture undergoes a solution-to-gel (sol-gel) phase transition. This retains ethanol near the injection site and significantly reduces the rate at which ethanol is cleared, by perfusion and/or diffusion into surrounding tissue. Then, injection rate was also optimized to maximize tumor retention of the mixture^27^. Notably, previous studies have shown that reducing fluid injection rate increases intratumoral injection efficacy^27^.

Our goal was to demonstrate proof-of-concept of enhanced ethanol ablation as an effective alternative to surgery for treatment of localized tumors, a method that can be suitable for resource-limited settings. Our injection design process addressed salient variables that govern biophysical ethanol retention and biological tumor regression: ethyl cellulose concentration in mixture with ethanol; injection needle gauge; injection rate; injection volume; and depth of needle insertion during injection. An agarose-based, poroelastic, tissue-mimicking mechanical phantom^30^ was used to analyze effects of ethyl cellulose concentration, injection volume and injection rate on fluid flow and distribution within a simulated tumor. A 27 Gauge needle was chosen as a balance between decreasing the pain associated with larger diameter needles and decreasing the injection pressure associated with smaller diameter needles. Injection depth was fixed to the center of the tumor (approximately 4 mm). Results indicated an optimal ethyl cellulose concentration (3%) and an optimum injection rate (10 mL/hr). This guided selection of injection variable values in ablation of chemically-induced epithelial tumors in the hamster cheek pouch model. As detailed below, results for tumor regression in response to the optimum conditions were markedly improved as compared to those for injection of pure ethanol.

## Materials and Methods

### Ethyl Cellulose-Ethanol Solution Preparation and Physical Characterization

Mixtures of ethyl cellulose (Sigma Aldrich, St. Louis, MO)-ethanol (200 proof, Koptec, King of Prussia, PA) and ethanol were prepared by stirring at room temperature. Ethyl cellulose concentration ranged from 0–6% (ethyl cellulose to ethanol, weight:weight). Concentrations greater than 6% ethyl cellulose were not evaluated because they did not fully dissolve in ethanol at room temperature. Solution viscosity was measured with a Brookfield Model RV-DVIII Ultra Programmable Rheometer (Brookfield Engineering, Middleboro, MA) at room temperature. The cone number was a CP-40. The range of shear rates tested spanned two decades to simulate the range of shear rates during injection. Data were only accepted for torques between 10 and 100% (this falls within the published sensitivity of the rheometer). To obtain a viscosity value, three viscosity measurements at each shear rate were averaged. Given that rheological behavior was Newtonian (viscosity was independent of applied shear rate), values across all shear rates were then averaged to characterize each ethyl cellulose concentration.

Upon contact with an aqueous environment, ethyl cellulose-ethanol solutions underwent a phase change, resulting in a stiffer material with higher viscoelasticity (rheological data not shown). We refer to this as “gel” for the sake of brevity, but emphasize that its full rheological characterization is not yet complete and will be explored, as needed, in future work. Gel formation was evaluated in relation to the ethyl cellulose concentration within and relative to the amount of water added to the original ethyl cellulose-ethanol solution. Gel formation rates were determined by adding 2 mL of ethyl cellulose-ethanol solutions to 10% fetal bovine serum (FBS, Sigma, St. Louis, MO) in phosphate buffered saline (PBS, Corning, Manassas, VA) in 15 mL or 50 mL centrifuge tubes. The FBS-PBS solution was added in volumes ranging from 0.5 to 35 mL. The centrifuge tubes were placed in a water bath at 37 °C (Branson 2510, Danbury, CT) and allowed to reach equilibrium for 10 minutes. To determine the amounts of ethyl cellulose within gels, they were placed in glass vials on a 120 °C hot plate for 6 hours until no liquid remained. Then the gels were removed from the vials and weighed. To calculate gel density, 3% ethyl cellulose-ethanol was added to an FBS-PBS solution (1:4 weight:weight), the gel was collected and weighed, and then placed in centrifuge tubes filled with water to determine the gel volume from the displaced fluid volume.

### Tumor-Mimicking Mechanical Phantoms

Agarose-based mechanical phantoms were used to evaluate distribution of injection volume *in vitro*. Such phantoms have been employed previously to optimize brain infusion protocols^30–32^. The phantoms were composed of 0.2% agarose (weight:weight, UltraPure Agarose, Invitrogen, Carlsbad, CA), which was stirred into deionized water over a hot plate for 3 hours until the solution was clear. This was then poured into 75 mL vials (20 dram polystyrene containers, Fisher Scientific, Hampton, NH), and allowed to cool at 4 °C for 24 hours to solidify. The phantoms were injected with test media, using 1 mL syringes affixed with 27 gauge needles using microtubing (Tygon Microtube Tubing, 0.25 mm inner diameter), and connected to a syringe pump (NE-300 Just Infusion Syringe Pump). Thus, these phantoms contained a poroelastic microstructure filled with aqueous medium (akin to tumors). Injections consisted of 50 µL of either pure ethanol or 3% ethyl cellulose-ethanol solutions, injected at rates ranging from 0.1 to 10 mL/hr, which were controlled by a syringe pump (NE-300 Just Infusion Syringe Pump). Higher injection rates of 100 mL/hr injections were performed manually, and measured with a stopwatch; this rate exceeded the capacity of the pump. Food dye was added to the injection media to enable visualization of distribution volume. This was defined as the volume which the injected solution occupied within the phantom. The depth for all injections was approximately 25 mm.

Thirty minutes after the onset of injections, images within the phantoms were obtained of the widest cross-sectional area of dye with a ruler in-plane. The phantom was then rotated 90 degrees and imaged again. MATLAB (version 2016a, Mathworks, Inc., Natick, Massachusetts) was used to optically segment the space occupied by the blue dye, and its volume was calculated using Equation (1):

1 $$Volume=\frac{4}{3}\ast Are{a}_{Cross-section}\ast Radiu{s}_{Orthogonal}$$

where $Are{a}_{cross-section}$ is the widest cross-sectional area and $Radiu{s}_{orthogonal}$ is the widest radius from the orthogonal perspective. Only dye 3.6 mm above or below the tip of the needle was measured, to mimic distribution within a spherical tumor 200 mm^3^ in volume; this is the average volume expected in our follow up *in vivo* experiments. Liquid extending above or below this tumor volume is likely to leak out of the tumor *in vivo* either into surrounding tissue or out along the injection pathway. Experiments for combinations of different injection rate and ethyl cellulose concentration were repeated seven times with injection volume held constant at 50 µL. Experiments for different combinations of injection volume and ethyl cellulose concentration were repeated 5–7 times, with the injection rate held constant at 10 mL/hr. Based on the resulting injection parameter space, we could deduce putative optimum values for ethyl cellulose concentration and injection rate.

### *In Vitro* Cell Viability Study

An initial evaluation of the cytotoxicity of the ethyl cellulose-ethanol injection mixture was performed using low passage HeLa human cervical carcinoma cells. Cells (obtained from the Duke University Cell Culture Facility, Durham, North Carolina) were maintained with Eagle’s minimum essential medium (MEM, Gibco, Carlsbad, California) supplemented with 10% (vol.) fetal bovine serum, 0.5% (vol.) penicillin, and 0.5% (vol.) streptomycin. Cells were passaged twice per week and maintained at 37 °C and 5% CO_2_. They were cultured in 12-well plates to 80% confluence.

Immediately before each experiment, cell medium was removed and 0.5 mL of fresh medium was added to each well. Next, 0.5 mL of either ethanol, 3% (weight:weight) ethyl cellulose (USP, Sigma Aldrich, Rockville, MD) in ethanol, or PBS (control) was added. The plates were then incubated at room temperature for 15 seconds; this exposure time to ethanol has been shown to induce substantial cell death^33^. The medium was then removed, and each well was rinsed twice with 1 mL of PBS and given 1 mL of fresh medium.

After all wells had been treated, each was rinsed one time with 1 mL of PBS, given 250 µL of 0.5% trypsin (Gibco, Carlsbad, CA), and returned to the incubator for 5 minutes. Once cells had lifted, 750 µL of medium was added, and the contents of the well were placed in individual vials and vortexed. Viability was then assessed with a trypan blue exclusion assay (Gibco, Carlsbad, CA) using a Countess Automated Cell Counter (Invitrogen, Carlsbad, CA). Two viability measurements (viability = live cell count/ total cell count) were obtained for each well and averaged. There were eight wells for each treatment group.

### Chemical Induction of Squamous Cell Carcinoma in the Hamster Cheek Pouch

Effects of injections on tumor regression were evaluated in the hamster cheek pouch model^34^. The animal study protocol was approved by the Duke University Institutional Animal Care and Use Committee and all studies were performed in accordance with relevant guidelines and regulations (Protocol Number A216-15-08**)**. All procedures were performed under isoflurane anesthesia. Hamsters were female Golden Syrian Hamsters between 100 and 150 grams. Tumors were induced through topical application of 7,12-dimethylbenz[a]anthracene (DMBA, Sigma-Aldrich)^34^. Three times a week, the buccal mucosa of each cheek pouch was everted and then stretched from the mouth. An area of approximately 5 cm^3^ was painted with a cotton swab dipped in the DMBA-mineral oil solution. The cheek pouches were painted until tumors developed, typically at around 22 weeks.

### Control Injections in Hamster Cheek Pouch Model: High-Volume Pure Ethanol

Pure ethanol injections at high volume were performed manually. The average initial tumor volume was 42 ± 34 µL (s.d.). 192 ± 106 µL (s.d) of pure ethanol mixed with food dye was injected into the center of the tumor. These injection volumes were controlled to achieve a total volume that was 3 to 4 times the tumor volume. Tumor volumes were measured with digital calipers before each injection and daily for 7 days thereafter. For tumors that did not respond and were still present after 7 days, repeat ablations were performed. These were treated as independent ablations (i.e., as though they were new tumors receiving their first ablations). Justification for treating repeat injections as independent injections is shown in Supplementary Figure 1 (tumors receiving a repeat injection behaved similarly to tumors receiving a first-time injection). Such repeats were only performed if the tumor volume had increased for two consecutive days after the 7-day observation period. Volumes were calculated by measuring the longest axis and the orthogonal axis and using Equation (2):

2 $$Volume=\frac{4}{3}\ast \pi \ast {(Radiu{s}_{long})}^{2}\ast Radiu{s}_{orthogonal}$$

where $Radiu{s}_{long}$ is the longest radius and $Radiu{s}_{orthogonal}$ is the orthogonal radius. Complete tumor regression was defined as the absence of any gross evidence of a tumor or raised lesion by visual examination. Twelve injections were performed in 6 hamsters. Six injections were repeat injections, and only one tumor was treated per hamster.

### Injection with Ethanol-Ethyl Cellulose: Varying Injection Rate and Ethyl Cellulose Concentration

**Figure 1:**
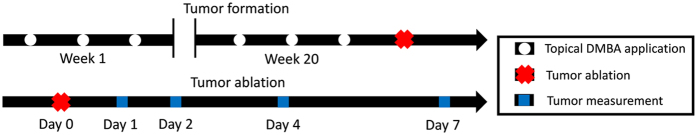
Study design for assessing effects of injection rate and ethyl cellulose concentration on therapeutic efficacy *in vivo*. Squamous cell carcinomas were induced in the hamster cheek pouch through topical application of DMBA 3X per week until tumors formed and reached a volume of 100 mm^3^ (approximately 20 weeks). Tumors were then injected with 50 µL of either ethanol or 3% ethyl cellulose-ethanol solution at a rate of 0.1, 1.0, 10 or 100 mL/hr. After ablation, tumor volume was measured at 1, 2, 4, and 7 days after treatment. For tumors that were still present after 7 days, repeat ablations were performed; these were treated as independent ablations if tumor volume had increased for two consecutive days after day 7.

Figure 1 illustrates the design of these experiments. For evaluation of varying injection rate and ethyl cellulose concentration, the average tumor volume was 195 ± 140 µL (mean ± s.d.). 50 µL of solution (either pure ethanol or 3% ethyl cellulose-ethanol) was injected into the center of the tumor. Injection volume was always less than tumor volume (about 25% of tumor volume, as compared to 400% in the control studies with pure ethanol that simulated current clinical practice). Injection rates were 0.1, 1.0 or 10 mL/hr (achieved using the syringe pump) or ~100 mL/hr (achieved manually). Notably, the rate of 10 mL/hr has been suggested as optimal for gene delivery into tumors^27^. Tumor volume was measured before injections, and at 1, 2, 4, and 7 days thereafter. For tumors that did not respond completely and were still present and growing after day 7, repeat ablations were performed (as for controls, above). These were treated as independent ablations (i.e., as though they were new tumors, as described above; justification for treating repeat injections as independent is shown in Supplementary Figure 1). Repeats were only performed if the tumor volume had increased for two consecutive days after the 7-day observation period. 36 total ablations were performed on 8 animals. 15 injections were repeat injections, and multiple tumors from each hamster were treated. The study design is illustrated in Fig. 1.

### Statistical Analysis

All statistical analysis was performed using R software (R Foundation for Statistical Computing, Vienna, Austria)^35^. For cell viability analysis, a parametric one-way analysis of variance (ANOVA) was used followed by a Tukey post-hoc test, to determine the relative cytotoxicities of ethanol, 3% ethyl cellulose-ethanol, and PBS. For both phantom distribution volume and *in vivo* normalized tumor volume analyses, non-parametric ANOVAs were performed (Kruskal-Wallis) followed by a non-parametric multiple comparisons test (Dunn’s test) because of the extent of variability. For injection volume, two-way parametric ANOVAs were performed, and the relationship between injection volume and distribution volume was fit to a linear model. The Pearson’s product moment correlation coefficient was calculated to assess the relationship between the phantom distribution volume and *in vivo* normalized tumor volume. Normalized tumor volume was calculated by dividing the volume at a given time point by the initial volume before treatment. Repeat injections were considered as independent injections. They did not perform significantly differently from first-time injections (Supplementary Figure 1). For *in vivo* experiments, tumors were randomly assigned to experimental groups. A significance level of p = 0.05 was applied to reject the null hypotheses in all analyses.

### Data Availability

The datasets generated during the current study are available in the Open Science Framework repository (found at: https://osf.io/582gd/).

## Results

### Physical properties of ethyl-cellulose and ethanol mixtures

**Figure 2:**
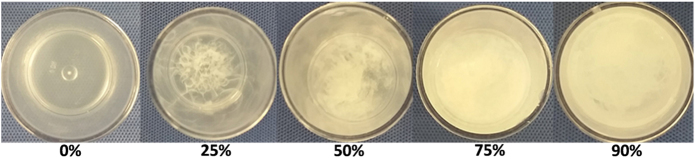
Ethyl cellulose-ethanol forms a gel upon exposure to water. A 3% ethyl cellulose-ethanol solution either alone (0%) or in solution with 20%, 50%, 75% or 90% water.

Figure 2 illustrates the phase transition of ethyl cellulose-ethanol solutions from a homogenous liquid solution (before addition of water) to the formation of a gel-phase upon increasing the water concentration. The density was measured to be 0.6 ± 0.08 (mean ± s.d., n = 4) g/mL. After weighing the gel, each specimen was heated until all the liquid evaporated, leaving only dried ethyl cellulose powder. This dry powder was then weighed and found to be 12.3% ± 3.0% (mean ± s.d., n = 27) of the original gel; this indicated that the vast majority of the gel was liquid. Since ethyl cellulose is ethanol-soluble and water-insoluble, the evaporated portion of the gel was likely to be primarily ethanol.

**Figure 3:**
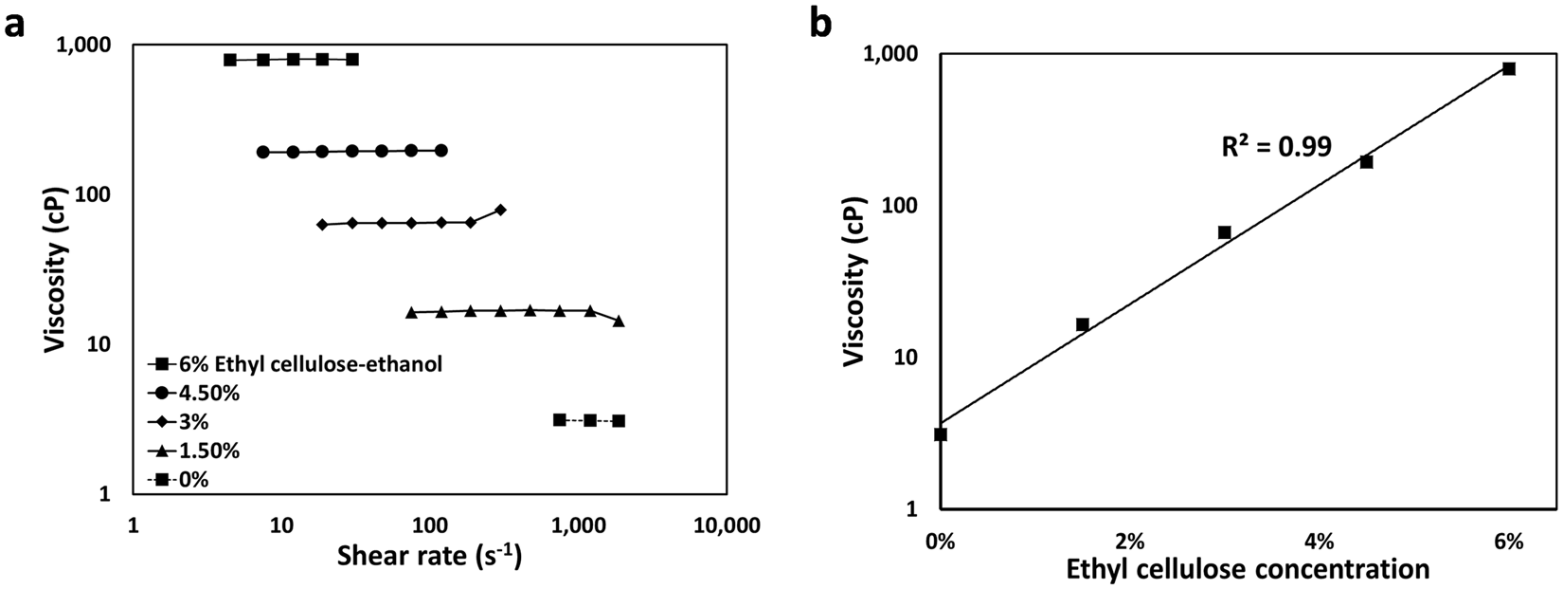
Ethanol viscosity increases with addition of ethyl cellulose. (**a**) Viscosity values were independent of shear rate. (**b**) Viscosity of ethanol increased with increasing ethyl cellulose concentration.

Ethyl cellulose-ethanol solutions ranging from 0 to 6% ethyl cellulose exhibited Newtonian behavior (viscosity was independent of shear rate, Fig. 3a). Viscosity increased exponentially with ethyl cellulose concentration (Fig. 3b).

**Figure 4:**
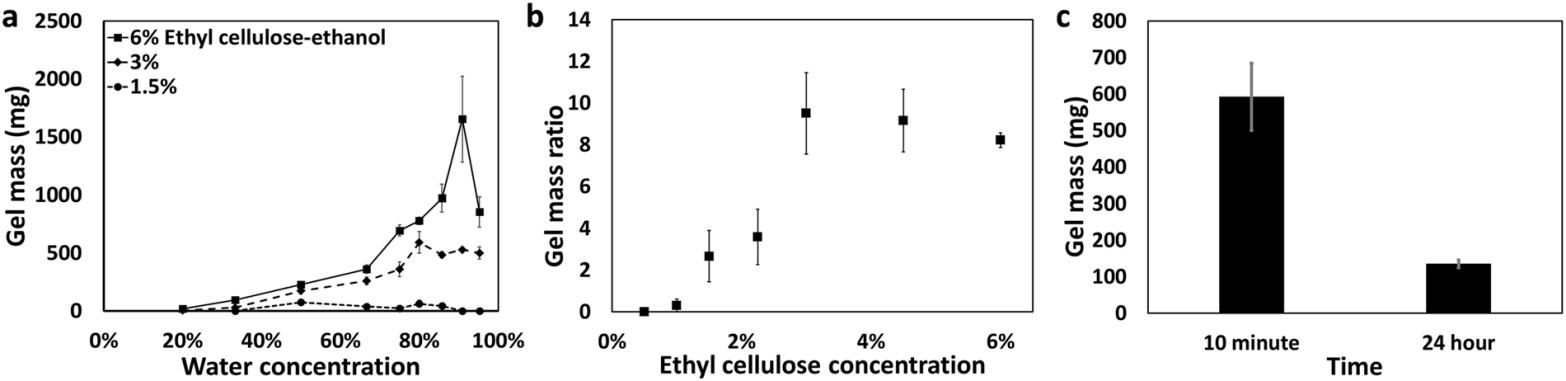
Gel mass is a function of water concentration, ethyl cellulose concentration and time. (**a**) Gel formation rates were a function of both on ethyl cellulose concentration and the ratio of water to ethanol (n = 3). (**b**) The gel mass ratio (i.e., the ratio of the gel mass to the mass of ethyl cellulose in the initial ethyl cellulose-ethanol solution) was a function of initial ethyl cellulose concentration. These solutions were 80% FBS-PBS (denoted “water”) and 20% ethyl cellulose-ethanol (n = 3). (**c**) Gel mass formation as a function of time (n = 3). All error bars depict standard error.

Gel formation is shown as a function of the percent FBS-PBS medium (referred to as “water”) that was added to the solution (Fig. 4a). Extent of gel formation initially increased with water concentration, peaked, and then decreased, with a modest drop for 3% ethyl cellulose-ethanol and a sharp decline for 6% ethyl cellulose-ethanol. The gel mass ratio is the mass of the gel divided by the mass of the ethyl cellulose powder initially in solution. This ratio (Fig. 4b) was used to determine the optimal formulation and represents a balance between increasing gel formation and decreasing potential systemic toxicity from excessive ethyl cellulose. The peak gel mass ratio occurred at a 3% ethyl cellulose concentration (Fig. 4b) when measured at 80% water concentration (a reasonable estimate for tumors)^36^. Gel mass originating from the mixture of 3% ethyl cellulose-ethanol and FBS-PBS (20% and 80%, respectively) at 37 °C was measured at 24 hours. The mass at 24 hours was 20.9 ± 30.8% (mean ± s.d., n = 4) of the mass measured at 10 minutes. This indicates that gels spontaneously degraded at physiological conditions (Fig. 4c).

### Distribution volume in phantoms is governed by injection parameters, and significantly increases with addition of ethyl cellulose

**Figure 5:**
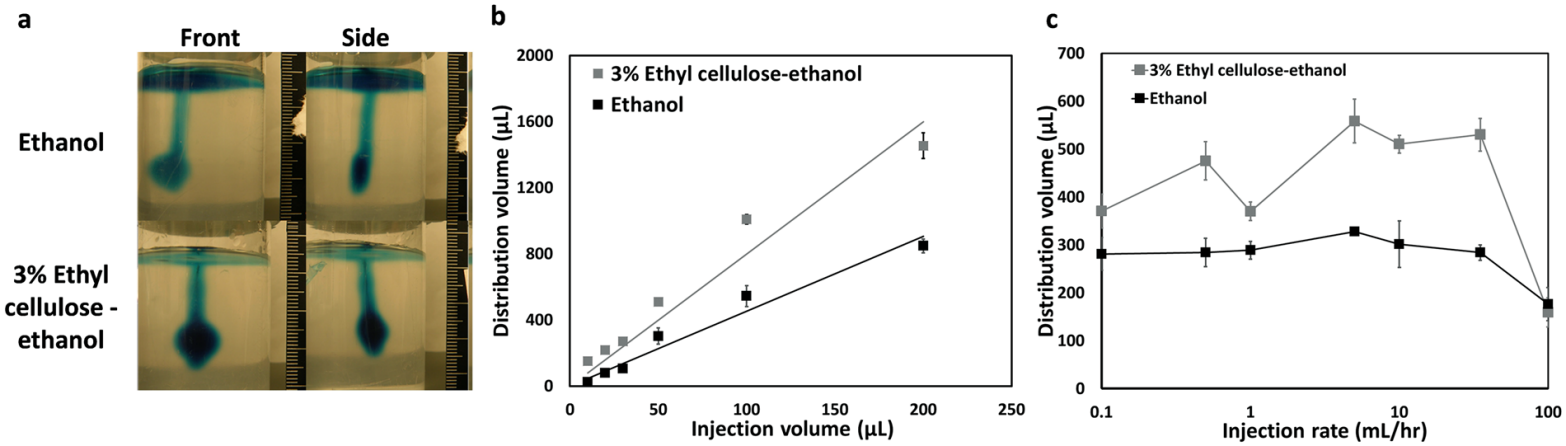
Distribution volume varies with injection rate, injection volume, and ethyl cellulose concentration. (**a**) Representative images of 50 μL of ethanol or 3% ethyl cellulose-ethanol injected into agarose tissue phantoms at 10 mL/hr. Images were taken from the perspective with the widest cross-sectional area (“Front”) and the orthogonal perspective (“Side”). Distribution volume was calculated from front and side images of each phantom, assuming that the distribution shape was an ellipsoid. (**b**) Distribution volume as a function of injection volume for 10 mL/hr injections of either ethanol or 3% ethyl cellulose-ethanol. The linear fits had slopes of 8.1 and 4.7, respectively. Each injection volume was performed 5 to 7 times. (**c**) Distribution volume as a function of injection rate for both pure ethanol and 3% ethyl cellulose-ethanol. Mean distribution volume of ethyl cellulose solution was higher than for ethanol alone (p < 0.01, exact p-value < 10^−7^, chi-square = 29.04, degrees of freedom = 1). Each injection rate was evaluated 7 times. All error bars depict standard error of the mean.

An underlying design goal in developing this tumor ablation methodology is to maximize distribution volume within the tumor while minimizing injection volume. This could increase therapeutic efficacy while decreasing potential toxicities, e.g. from excess ethanol that might leak into tissue surrounding the tumor. Experiments with the phantoms provided biophysically relevant insights about achieving that goal. Representative images acquired from two orthogonal perspectives of a phantom are shown in Fig. 5a. Dependence of distribution volume on injection volume (for injection rate of 10 mL/hr and injection volume ≤200 µL) is shown in Fig. 5b. Linear relationships were seen for both pure ethanol (R^2^ = 0.93, exact p-value = <2^−16^, t-Value = 19.41, degrees of freedom = 29) and 3% ethyl cellulose-ethanol (R^2^ = 0.97, exact p-value < 2^−16^, t-value = 29.89, degrees of freedom = 32). The slope for the 3% ethyl cellulose-ethanol mixture was nearly double that for ethanol (8.1 vs. 4.7, exact p-value = 1.6E-12, F-value = 79.42, degrees of freedom = 1). This was likely due to the increased backflow of lower viscosity ethanol around the injection needle for the latter. Backflow denotes the flow of the injection solution upwards along the needle and back out of the phantom. Distribution volume vs. injection rate (for a 50 µL injection volume) is shown in Fig. 5c. As averaged over all injection rates, distribution volume for 3% ethyl cellulose-ethanol was 421 ± 350 mm^3^ (mean ± s.d., n = 49) vs. 278 ± 247 mm^3^ (mean ± s.d., n = 49) for pure ethanol. Despite the high variability, these values are significantly different (p < 0.01, exact p-value < 10^−7^, chi-square = 29.04, degrees of freedom = 1).

### Injection of pure ethanol exhibits low therapeutic efficacy

**Figure 6:**
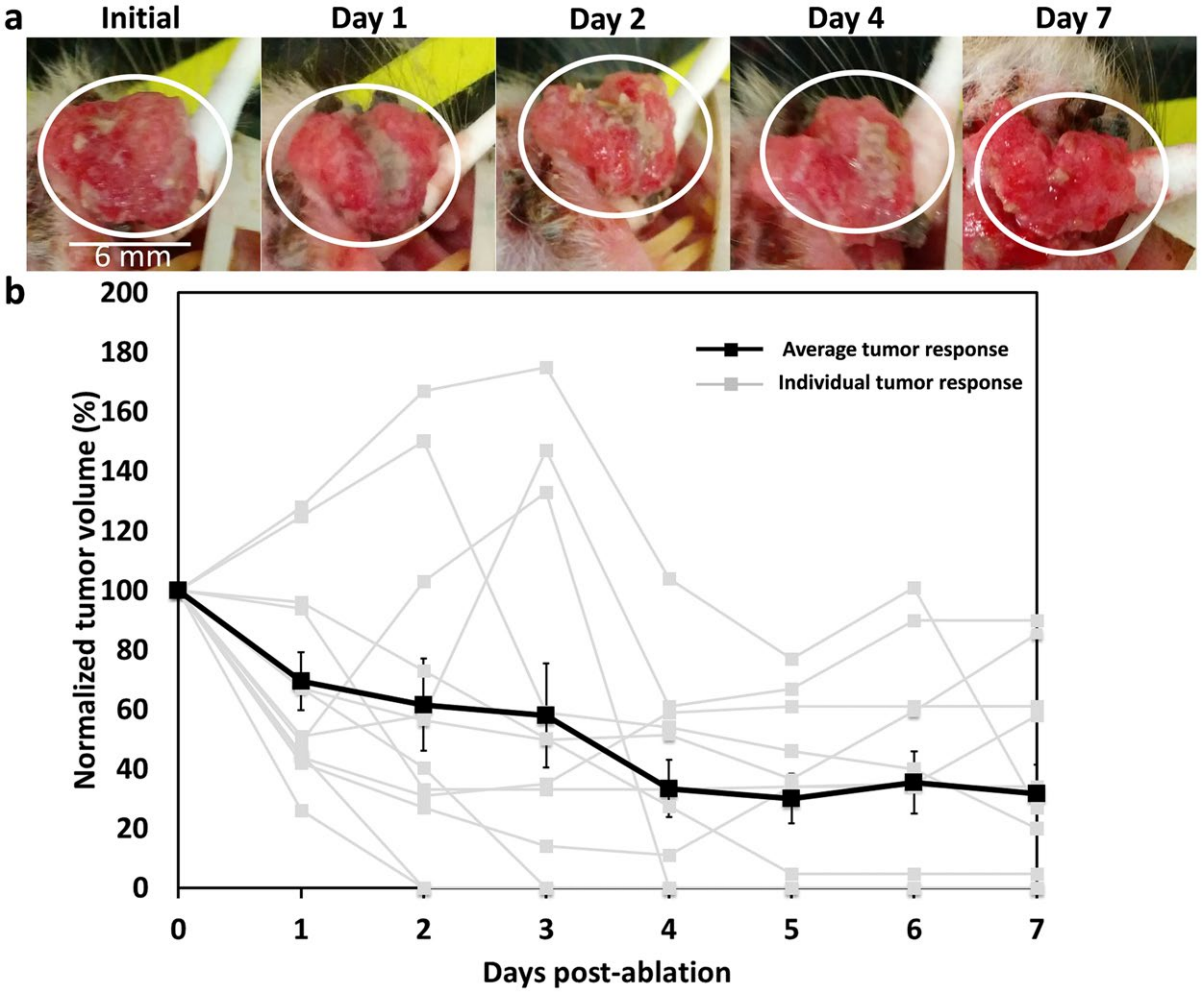
High-dosage manual ethanol ablation treatment of epithelial tumors is ineffective. (**a**) Representative images show typical tumor response over time, in which complete regression does not occur. Scale bar is approximately 6 mm. (**b**) 12 squamous cell carcinoma tumors were ablated and the tumor volume was measured over 7 days. At day 7, the average tumor volume was 32% of the initial volume and 4 out of 12 tumors had responded completely with no sign of a tumor. Therapeutic efficacy is quantified as normalized tumor volume, which is tumor volume as a percentage of its Day 0 value. Gray traces represent individual tumor responses and error bars depict standard errors of the means.

Figure 6 shows representative images of tumor response to conventional ethanol ablation (intratumoral injection of pure ethanol by hand) and the average reductions in tumor volume over a 7-day period. The needle was placed approximately 4 mm deep into the center of the tumor. The average initial tumor volume was 42 ± 34 µL (mean ± s.d.); and the average volume of ethanol injected was 192 ± 106 µL (mean ± s.d.), which is approximately four times the tumor volume. The needle was placed approximately 4 mm deep into the center of the tumor. For most injections, some necrosis was visible and the overall tumor volume decreased, but complete response was not consistently achieved (Fig. 6a). On average the tumor volume decreased to 32 ± 34% (mean ± s.d.) of the initial volume by day 7 (Fig. 6b). Of the 12 tumors ablated, 4 regressed completely and had no visible lesions at day 7.

### Moderate injection rates and increased ethyl cellulose concentration improve the therapeutic efficacy of ethanol ablation without altering solution cytotoxicity

**Figure 7:**
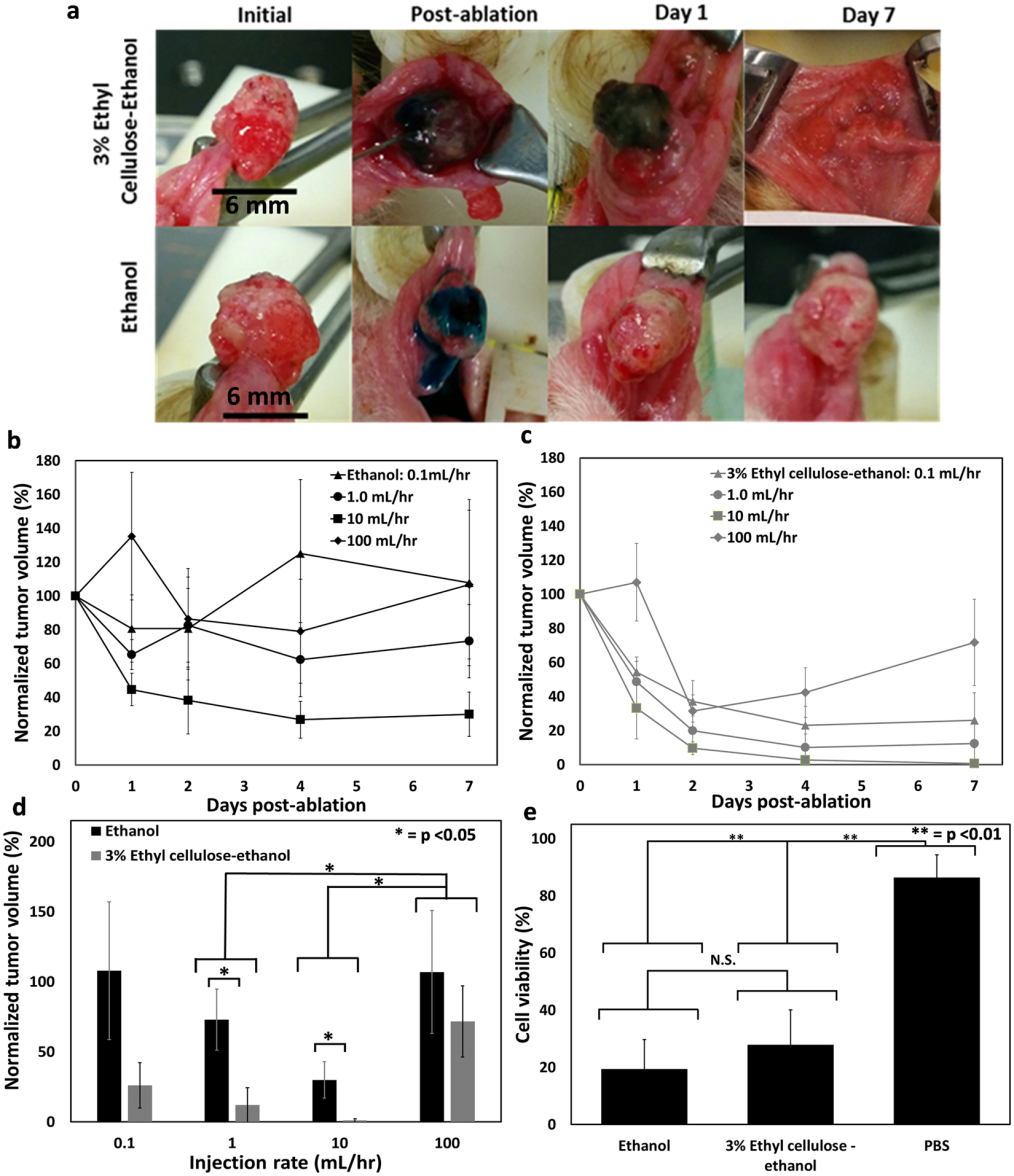
Therapeutic efficacy of enhanced ethanol ablation depends on ethyl cellulose concentration and rate. (**a**). Representative images of tumors for 3% ethyl cellulose-ethanol (top) and ethanol (bottom) injected at 10 mL/hr. Scale bar is 6 mm. Normalized tumor volume is defined as tumor volume at specified time point divided by initial volume. Normalized volume measured up to day 7 is shown for ethanol (**b**) and 3% ethyl cellulose-ethanol (**c**). Average initial tumor volume was 195 ± 140 µL (mean ± s.d) and injected solution volume was kept constant at 50 µL. (**d**) Normalized tumor volumes at day 7 for each ethyl cellulose concentration – injection rate combination. 3% ethyl cellulose-ethanol injections were more effective than ethanol injections (p < 0.01, exact p-value = 9.42 × 10^−4^, chi-square = 10.938, degrees of freedom = 1). Each condition (injection rate and ethyl cellulose concentration) was evaluated with 5 separate tumor ablations, except for 3% ethyl cellulose-ethanol injections at 0.1, 1.0 and 10 mL/hr, which were repeated 7 times. (**e**) There were no significant differences in cytotoxicity, as measured by cell viability, between ethanol and 3% ethyl cellulose-ethanol solutions, but both were substantially more cytotoxic than PBS. Each cytotoxicity condition was repeated 8 times. All error bars depict standard error.

To evaluate the interacting effects of ethyl cellulose concentration and injection rate on therapeutic efficacy *in vivo*, ablations were performed on chemically-induced squamous cell carcinoma tumors in the hamster cheek pouch at injection rates of 0.1, 1.0, 10, and 100 mL/hr, using either 3% ethyl cellulose-ethanol or pure ethanol. Therapeutic efficacy was again quantified as normalized tumor volume reduction which is defined as tumor volume 7 days after the treatment divided by the initial tumor volume (before treatment). Given the average tumor size of approximately 200 μL, the injection volume was set at 50 μL since this is the minimum volume at which both pure ethanol and 3% ethyl cellulose would fill an entire 200 μL tumor based on phantom results (see Fig. 5b). Thus, the injected volumes of both ethanol and ethyl cellulose-ethanol were reduced from four times the tumor volume (used for ethanol, Fig. 6) to a one-fourth of the tumor volume. The needle was placed approximately 4 mm deep in the center of the tumor. For each testing condition (injection rate/ethyl cellulose concentration), between 5 and 7 tumors were ablated. Representative images (shown in Fig. 7a) illustrate the tendency of pure ethanol injections to immediately leak out of the tumor; this phenomenon was observed less frequently during 3% ethyl cellulose-ethanol injections. The time courses of tumor response to ethanol or ethyl cellulose-ethanol ablation are shown in Fig. 7b and c, respectively. Individual tumor responses are shown in Supplementary Figure 2. Normalized tumor volume at day 7 depended on both injection rate and ethyl cellulose concentration (Fig. 7d). At day 7, the normalized tumor volume across all injection rates was 25 ± 43% (mean ± s.d., n = 26) for 3% ethyl cellulose-ethanol and 79 ± 79% (mean ± s.d., n = 20) for pure ethanol. The high variability in these average values likely reflects the varying properties of the tumors and their varying biological responses to the injection rates. They are significantly different (p < 0.01, exact p-value = 9.42 × 10^−4^, chi-square = 10.938, degrees of freedom = 1). The 10 mL/hr rate had the lowest overall mean normalized tumor volume of 13 ± 23% (mean ± s.d., n = 12) as compared to manual injections, which had a mean normalized volume of 89 ± 78% (mean ± s.d., n = 10); those values are significantly different (p < 0.05, exact p-value = 0.022, chi-square = 8.34, degrees of freedom = 3). Of the 7 tumors injected with 3% ethyl cellulose at 10 mL/hr, 6 out of 7 completely regressed by day 7, and 7 out of 7 completely regressed by day 8. Of the 5 tumors injected with pure ethanol at 10 mL/hr, 0 out of 5 regressed completely. These findings demonstrated that the combination of a moderate injection rate with the addition of ethyl cellulose provided the greatest treatment efficacy. To determine whether the differences in tumor regression were due to increased cytotoxicity of ethyl cellulose-ethanol, a cell viability study comparing the two different injection media was performed. No significant difference was found in the viability of HeLa cells treated with ethanol vs. 3% ethyl cellulose-ethanol [19 ± 10% and 28 ± 12% (mean + s.d., n = 8), respectively, exact p-value = 0.249, degrees of freedom = 14] as measured by a trypan blue exclusion assay (Fig. 7e).

### Therapeutic efficacy of ethyl cellulose-ethanol ablation *in vivo* correlates with distribution volume in tissue phantoms *in vitro* as a function of injection rate

*In vivo*, 3% ethyl cellulose-ethanol injections were more effective than ethanol injections as quantified by normalized tumor volume at day 7 (Fig. 7). We hypothesized that therapeutic efficacy would be strongly dependent on the distribution volume of the injected solution within the tumor, which governs the number of malignant cells contacted. Notably, 3% ethyl cellulose-ethanol injections achieved significantly higher distribution volumes than ethanol injections in our mechanical phantoms (Fig. 5).

**Figure 8:**
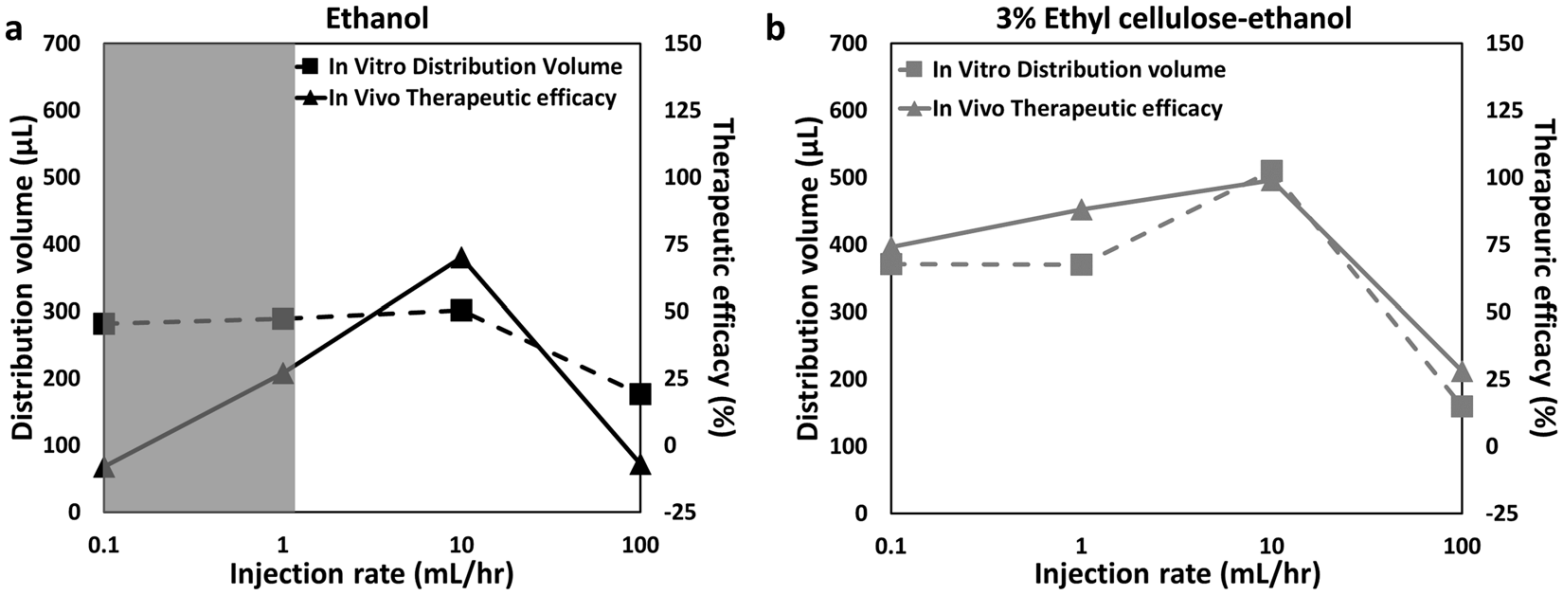
*In vivo* tumor ablations and *in vitro* tissue phantom distribution volumes have comparable results. *In vivo* therapeutic efficacy is defined as (1 – normalized tumor volume at day 7). *In vitro* tissue phantom distribution volumes were measured at rates used for the *in vivo* tumor ablations. (**a**) For ethanol injection, therapeutic efficacy did not correlate with distribution volume in injection rate-matched phantoms (Pearson’s correlation coefficient = 0.62, exact p-value = 0.382, t-value = −1.112, degrees of freedom = 2). The shaded area represents the injection rates that are approximately equal to the vascular clearance rates, likely reducing the accumulation rate within the tumor. (**b**) In contrast, for ethyl cellulose, the correlation was substantially higher (Pearson’s correlation coefficient = 0.96, exact p-value = 0.0352, t-value = 5.184, degrees of freedom = 2).

There was a strong correlation between *in vivo* therapeutic efficacy (quantified here as 1- normalized tumor volume at day 7) and injection rate for ethyl cellulose-ethanol (Fig. 8b, Pearson’s coefficient = 0.96, p < 0.05, exact p-value = 0.0352, t-value = 5.184, degrees of freedom = 2). The correlation between efficacy and distribution volume was not significant for ethanol only solution (Fig. 8a, Pearson’s coefficient = 0.62, exact p-value = 0.382, t-value = −1.112, degrees of freedom = 2). This may have been due to the fact that the low viscosity of ethanol causes it to be cleared (by leakage and/or vascular diffusion) at rates comparable to slower injection rates (0.1 and 1.0 mL/hr, shaded gray); as a result, there would be little accumulation within a tumor. Such vascular clearance was not embodied in the mechanical phantoms, causing divergence of the *in vivo* animal and *in vitro* phantom results (shaded area in Fig. 8a).

## Discussion

Although surgery is at the foundation of tumor treatment in developed countries, nine of ten people in developing countries do not have access to it due to lack of trained personnel and/or necessary equipment^4, 7^. The objective of this study was to develop an effective, low-cost alternative to surgery for tumor treatment, that eliminates the needs for specialized equipment, electricity, and/or a sterile environment. This was pursued through modification of ethanol ablation, an existing tumor ablation procedure entailing direct injection of ethanol into a tumor to induce necrosis. That technique was pioneered as a surgical alternative for treating hepatocellular carcinomas, with 5-year survival rates comparable to surgical resection^17^. It has achieved widespread success in applications to multiple tumor types^16, 19–22^. Conventional ethanol ablation however, requires injection of large volumes of ethanol and/or repeat injections^28^. Further, it has diminished efficacy in treating tumors not surrounded by fibrous capsules^23^.

Our enhanced ethanol ablation strategy retains ethanol at the injection site via addition of ethyl cellulose, a water-insoluble and ethanol-soluble cellulose-derivative. During injection into a tumor, this mixture undergoes a solution-to-gel (sol-gel) phase transition, as it is exposed to water. We first determined the optimal formulation based on gel formation rates as a function of water and ethyl cellulose concentration. Then we optimized the injection rate to maximize retained injection volume, using tissue-simulating phantoms. Finally we evaluated the method *in vivo*, using chemically-induced squamous cell carcinoma tumors in the hamster cheek pouch^34^. This animal model provides a high degree of similarity to human primary tumors, particularly those of the cervix and head and neck^37^. Historically, ethanol ablation has performed substantially worse in tumors not surrounded by fibrous capsules^23^. Thus, treatment of protruding epithelial tumors presented a challenge to our procedure, since they are not surrounded by tissue. This renders them highly susceptible to leakage of the injected solution away from the injection site. As expected, conventional high-volume ethanol ablation was ineffective and induced complete regression in only 33% of the tumors in our study (Fig. 6). In comparison, the enhanced ethyl cellulose-ethanol ablation procedure successfully induced regression in 100% of tumors (Fig. 7) despite a reduction in injection volume from 400% to 25% of tumor volume. This new approach is clearly more effective in reducing tumor volume and in reducing the overall injection volume of ethanol.

The addition of ethyl cellulose significantly increased therapeutic efficacy, we suggest, for two reasons: (1) the decrease of injected liquid that leaked out of the tumor, likely due to increased viscosity of the injection medium; and (2) the increased retention within the tumor due to the formation of an ethanol-based gel upon exposure to the aqueous tumor environment. Our tissue-mimicking mechanical phantom experiments showed that ethyl cellulose-ethanol solutions exhibited lower backflow rates (up the injection pathway and out of the phantom) than pure ethanol (Fig. 5). The reduction of backflow effectively increased the volume of solution delivered to the tumor. In treatment of percutaneous tumors (e.g. liver or breast), this reduction in backflow would decrease contact with, and therefore potential damage to, surrounding tissue. Further, ethyl cellulose-ethanol gel formation in an aqueous medium enabled a lower volume of ethanol) to access and treat the entire tumor volume, by increasing retention within the tumor. Further, it is possible that this enhanced retention of ethyl cellulose-ethanol slowed clearance into the vasculature, which could diminish unintended side effects of ethanol on surrounding tissue.

We analyzed the conjoint effects of injection rate and ethyl cellulose concentration on therapeutic efficacy (Fig. 7). This expanded upon studies of conventional ethanol ablation of tumors and ethyl cellulose-ethanol ablation of venous malformations, which described injection rate as “slow”^24, 38, 39^ or did not specify injection rate^17^. Our data from *in vitro* and *in vivo* models extend previous reports of the non-linear relationships between intratumoral injection rate and retention^27^. Lowering injection rate decreases pressure, which may decrease both the likelihood of backflow and also of crack formation in the heterogenous tumor structure that may induce leakage into surrounding tissue^40^. Mitigating these sources of leakage will result in increased tumor retention of ethanol and, therefore, increased therapeutic efficacy. Low-rate injections (0.1 mL/hr), notably, were less effective than medium-rate injections (10 mL/hr) for both ethanol and ethyl cellulose-ethanol solutions. A similar trend was observed for the distribution volume of ethyl cellulose-ethanol, but not pure ethanol, as measured in the tissue-mimicking phantom. A possible explanation is that low-rate ethyl cellulose-ethanol injections do not generate enough pressure to push the newly-formed gel through the porous structures of the tissue-mimicking phantom or the tumor. In contrast, since ethanol does not induce gel formation upon contact with the aqueous environment, low-rate ethanol injections had a comparable distribution volume to medium-rate injections. *In vivo*, these low-rate ethanol injections may have been ineffective because accumulation in the tumor decreased as the injection rate approached the vascular clearance rate. Of course, there was no vascular clearance in the phantoms. Such clearance may have contributed to poorer performance by ethanol within tumors, as seen in Fig. 8a.

Ethyl cellulose has previously been used to treat venous malformations. Here, injections were performed manually and 5.88% ethyl cellulose-ethanol was used. This led to significantly less pain than for pure ethanol injection, without systemic side effects^38, 39^. Our results expand on the initial success of those encouraging pilot clinical studies by focusing on solid tumors rather than vasculature. While further research is required to understand the mechanism of gel degradation *in vivo*, our *in vitro* results demonstrate that gel mass decreases 79% after 24 hours at 37 °C (Fig. 3g). Previous clinical studies that report spontaneous resorption of ethyl cellulose corroborate these findings^38, 39^. Degradation and resorption of ethyl cellulose would limit vascular occlusion in other parts of the body.

Ethyl cellulose is currently approved by the US FDA as a food additive and costs less than $0.50/gram. Even with a sixteen-fold reduction in injection volume from approximately 400% to 25% of tumor volume, the enhanced treatment by ethanol-ethyl cellulose was still more effective than conventional ethanol ablation. Such reduction in the injection volume, coupled with the linear relationship between injection volume and distribution volume *in vitro* (Fig. 5b), suggests that enhanced ethanol ablation can be modified to treat larger tumors, inducing a larger volume of necrosis by increasing the injection volume. In contrast, the volume of necrosis induced by thermal ablative techniques is limited by the amount of energy that can be deposited without boiling tissue (and therefore reducing thermal conductivity) and the loss of heat due to tissue perfusion^41^. In this context, enhanced ethanol ablation is an attractive tumor ablation modality. Since there was a high level of agreement between *in vitro* distribution volume and *in vivo* tumor volume reduction for ethyl cellulose-ethanol injections in our study, we propose that use of mechanical phantoms can contribute to expanded optimization of enhanced ethanol ablation procedures.

Although we have demonstrated the efficacy of enhanced ethanol ablation in the treatment of squamous cell carcinomas in the hamster cheek pouch, there are several limitations to this study. First, the use of a chemically-induced tumor model (in which spontaneous tumors arising in sites adjacent to a treated site would be indistinguishable from the original tumor) precluded the possibility of any long-term monitoring of tumor recurrence. Second, a single animal tumor model was utilized. Further study in other tumor models is needed to investigate such recurrence, and demonstrate therapeutic efficacy more broadly. Since this study was a proof-of-concept, sample sizes were relatively low. Although other studies have shown systemic safety of ethanol ablation in a variety of applications^18, 20–26^, more research is needed to investigate the possibility of damage to surrounding tissue. Leakage of ethanol and nearby necrosis should be minimized, and details may be specific to tumor types. Although ethyl cellulose-ethanol has been used in the treatment of venous malformations^38^, further safety evaluations of injections to tumors are clearly necessary. The ethanol-ethyl cellulose-water system has shown promise; but its more complete characterization will inform optimization. For example, better understanding of physicochemical details of the sol-gel transition, e.g. involving pressure and temperature, will benefit from improved discrimination between the sol and gel phases. The mechanical phantoms proved helpful in this initial study. Follow up use of them can improve details of their poroelastic structures and properties, and thus improve their role in the design process for enhanced ethanol ablation.

Overall, our results suggest that this enhanced version of ethanol ablation could be useful in the treatment of a number of malignancies. Notably, breast cancer is the leading cause of cancer-related deaths in low-income countries^1^, and conventional ethanol ablation has been used to treat tumors up to 5 cm^23^. Thus, enhanced ethanol ablation might be suitable for treatment of palpable breast tumors. Application to cervical precancerous lesions is another example. Cryotherapy, the standard-of-care for cervical precancerous lesion treatment in resource-limited settings, is incapable of consistently treating advanced precancerous lesions^15^ and requires hard-to-supply consumables^13^. Enhanced ethanol ablation is less expensive, does not require these consumables, and can treat a larger volume of tissue. Given the general lack of accessibility to surgery or alternative tumor treatments in developing countries^7^ and the promising results presented in this study, enhanced ethanol ablation is a promising method to meet the unmet clinical need of rising cancer mortality that challenges healthcare systems in developing countries.

## Electronic supplementary material

Supplementary Information

## References

[1] Ferlay J (2015). Cancer incidence and mortality worldwide: sources, methods and major patterns in GLOBOCAN 2012. Int J Cancer.

[2] Torre LA (2015). Global cancer statistics, 2012. CA Cancer J Clin.

[3] Preker, A. S., McKee, M., Mitchell, A. & Wilbulpolprasert, S. In *Disease Control Priorities in Developing Countries* (eds D. T. Jamison *et al*.) (2006).

[4] Kingham TP (2013). Treatment of cancer in sub-Saharan Africa. Lancet Oncol.

[5] Kushner AL (2010). Addressing the Millennium Development Goals from a surgical perspective: essential surgery and anesthesia in 8 low- and middle-income countries. Arch Surg.

[6] Hagopian A, Thompson MJ, Fordyce M, Johnson KE, Hart LG (2004). The migration of physicians from sub-Saharan Africa to the United States of America: measures of the African brain drain. Hum Resour Health.

[7] Meara JG (2015). Global Surgery 2030: evidence and solutions for achieving health, welfare, and economic development. Lancet.

[8] Martin RC, Scoggins CR, McMasters KM (2010). Safety and efficacy of microwave ablation of hepatic tumors: a prospective review of a 5-year experience. Ann Surg Oncol.

[9] Gervais DA, McGovern FJ, Arellano RS, McDougal WS, Mueller PR (2005). Radiofrequency ablation of renal cell carcinoma: part 1, Indications, results, and role in patient management over a 6-year period and ablation of 100 tumors. AJR Am J Roentgenol.

[10] Duncan ID (1983). The Semm cold coagulator in the management of cervical intraepithelial neoplasia. Clin Obstet Gynecol.

[11] Duncan I (2014). Cold coagulation in the treatment of cervical intraepithelial neoplasia. Bjog-Int J Obstet Gy.

[12] Chen J (2016). Industry-academic partnerships: an approach to accelerate innovation. J Surg Res.

[13] PATH. *Treatment Technologies for Precancerous Cervical Lesions in Low-Resource Settings: Review and Evaluation*, 2013.

[14] Tsu VD, Jeronimo J, Anderson BO (2013). Why the time is right to tackle breast and cervical cancer in low-resource settings. Bull World Health Organ.

[15] Mariategui J, Santos C, Taxa L, Jeronimo J, Castle PE (2008). Comparison of depth of necrosis achieved by CO_2_- and N_2_O-cryotherapy. Int J Gynaecol Obstet.

[16] Sugiura N., Ohto, T. K., Okuda, M. & Hirooka, K. N. Percutaneous intratumoral injection of ethanol under ultrasound imaging for treatment of small hepatocellular carcinoma. *Acta Hepatol Jpn* **21** (1983).

[17] Ryu M (1997). Therapeutic results of resection, transcatheter arterial embolization and percutaneous transhepatic ethanol injection in 3225 patients with hepatocellular carcinoma: a retrospective multicenter study. Jpn J Clin Oncol.

[18] Sorajja P (2008). Outcome of alcohol septal ablation for obstructive hypertrophic cardiomyopathy. Circulation.

[19] Solbiati L (1985). Percutaneous ethanol injection of parathyroid tumors under US guidance: treatment for secondary hyperparathyroidism. Radiology.

[20] Jurgensen C (2006). EUS-guided alcohol ablation of an insulinoma. Gastrointest Endosc.

[21] Artifon EL (2007). EUS-guided alcohol ablation of left adrenal metastasis from non-small-cell lung carcinoma. Gastrointest Endosc.

[22] DeWitt J, Mohamadnejad M (2011). EUS-guided alcohol ablation of metastatic pelvic lymph nodes after endoscopic resection of polypoid rectal cancer: the need for long-term surveillance. Gastrointest Endosc.

[23] Kuang M (2009). Ethanol ablation of hepatocellular carcinoma Up to 5.0 cm by using a multipronged injection needle with high-dose strategy. Radiology.

[24] Ebara M (2005). Percutaneous ethanol injection for small hepatocellular carcinoma: therapeutic efficacy based on 20-year observation. J Hepatol.

[25] Huang GT (2005). Percutaneous ethanol injection versus surgical resection for the treatment of small hepatocellular carcinoma: a prospective study. Ann Surg.

[26] Heilo A (2011). Efficacy of ultrasound-guided percutaneous ethanol injection treatment in patients with a limited number of metastatic cervical lymph nodes from papillary thyroid carcinoma. J Clin Endocrinol Metab.

[27] Wang Y, Wang H, Li CY, Yuan F (2006). Effects of rate, volume, and dose of intratumoral infusion on virus dissemination in local gene delivery. Mol Cancer Ther.

[28] Gelczer RK, Charboneau JW, Hussain S, Brown DL (1998). Complications of percutaneous ethanol ablation. J Ultrasound Med.

[29] Wang Y, Liu S, Li CY, Yuan F (2005). A novel method for viral gene delivery in solid tumors. Cancer Res.

[30] Pomfret R, Miranpuri G, Sillay K (2013). The substitute brain and the potential of the gel model. Ann Neurosci.

[31] Chen ZJ (2004). A realistic brain tissue phantom for intraparenchymal infusion studies. J Neurosurg.

[32] Chen ZJ, Broaddus WC, Viswanathan RR, Raghavan R, Gillies GT (2002). Intraparenchymal drug delivery via positive-pressure infusion: experimental and modeling studies of poroelasticity in brain phantom gels. IEEE Trans Biomed Eng.

[33] Tapani E, Taavitsainen M, Lindros K, Vehmas T, Lehtonen E (1996). Toxicity of ethanol in low concentrations. Experimental evaluation in cell culture. Acta Radiol.

[34] Burns RA, Klaunig JE, Shulok JR, Davis WJ, Goldblatt PJ (1986). Tumor-localizing and photosensitizing properties of hematoporphyrin derivative in hamster buccal pouch carcinoma. Oral Surg Oral Med Oral Pathol.

[35] Team, R. C. R: A language and environment for statistical computing. R Foundation for Statistical Computing, Vienna, Austria. (2016).

[36] Kiricuta IC, Simplaceanu V (1975). Tissue water content and nuclear magnetic resonance in normal and tumor tissues. Cancer Res.

[37] Liu Y (2015). Mammalian models of chemically induced primary malignancies exploitable for imaging-based preclinical theragnostic research. Quant Imaging Med Surg.

[38] Sannier K (2004). A new sclerosing agent in the treatment of venous malformations. Study on 23 cases. Interv Neuroradiol.

[39] Dompmartin A, Vikkula M, Boon LM (2010). Venous malformation: update on aetiopathogenesis, diagnosis and management. Phlebology.

[40] Netti PA, Berk DA, Swartz MA, Grodzinsky AJ, Jain RK (2000). Role of extracellular matrix assembly in interstitial transport in solid tumors. Cancer Research.

[41] Goldberg SN, Gazelle GS, Mueller PR (2000). Thermal ablation therapy for focal malignancy: a unified approach to underlying principles, techniques, and diagnostic imaging guidance. AJR Am J Roentgenol.

