# Supplementary material for: Development of enhanced ethanol ablation as an alternative to surgery in treatment of superficial solid tumors
Source: Sci Rep. 2017 Aug 18;7:8750. doi: 10.1038/s41598-017-09371-2 (PMC5562881; doi:10.1038/s41598-017-09371-2)
Supplement: Supplementary file 1 — Supplementary Information [file 41598_2017_9371_MOESM1_ESM.pdf]

Development of enhanced ethanol ablation as an alternative to surgery in treatment of superficial solid tumors

Robert Morhard[1], Corrine Nief[1], Carlos Barrero Castedo[1], Fangyao Hu[1], Megan Madonna[1], Jenna L. Mueller[1], Mark W. Dewhirst[2], David F. Katz[1,3], Nirmala Ramanujam[1,3,4,5]

[1]Department of Biomedical Engineering, Duke University, Durham, North Carolina, United States of America, [2]Department of Radiation Oncology, Duke University, Durham, North Carolina, United States of America, [3]Department of Obstetrics and Gynecology, Duke University, [4]Department of Pharmacology and Cancer Biology, Duke University, Durham, North Carolina, United States of America, [5]Duke Global Health Institute, Duke University, Durham, North Carolina, United States of America

*Corresponding author: robert.morhard@duke.edu (RM)

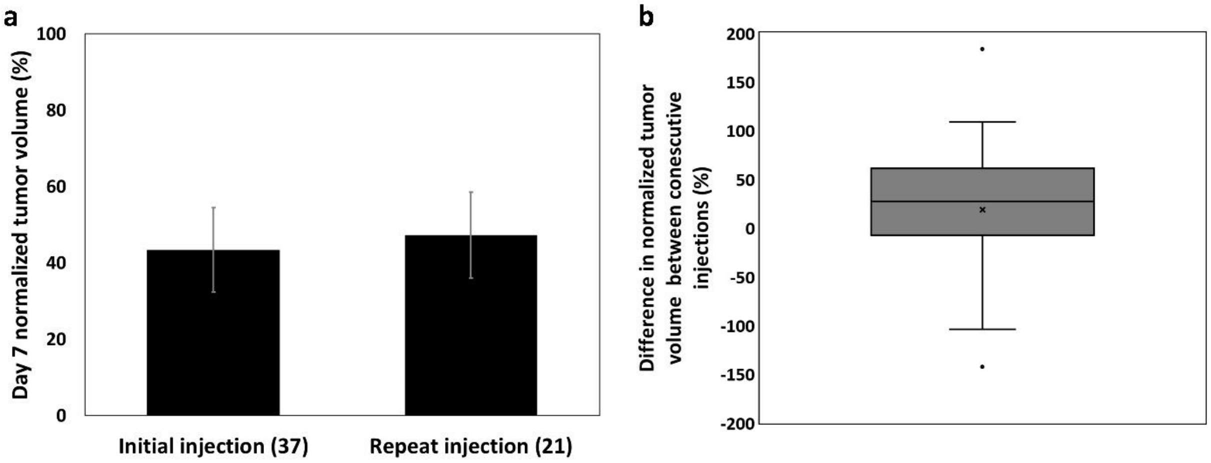

Supplementary Figure 1: a) In both the first ablation study of manual pure ethanol injection and the second study with controlled injection rate and ethyl cellulose concentration there were 21 total repeat injections. The average tumor volume at day 7 for an initial injection was 0.43 and for a repeat injection was 0.47. Error bars depict standard error. **b)** The difference in normalized tumor volume reduction between sequential injections for repeat injections is calculated by taking the difference in normalized tumor volume reduction by a repeat injection and the preceding injection. The average difference is 20 ± 75 (standard deviation) which is not statistically different than 0. The difference is larger than in A) because initial injections that resulted in total tumor regression could not be ablated again and are therefore not included in B. The distribution is shown above in the box and whisker plot. The lack of any statistical difference between repeat and initial injections justifies their categorization as independent.

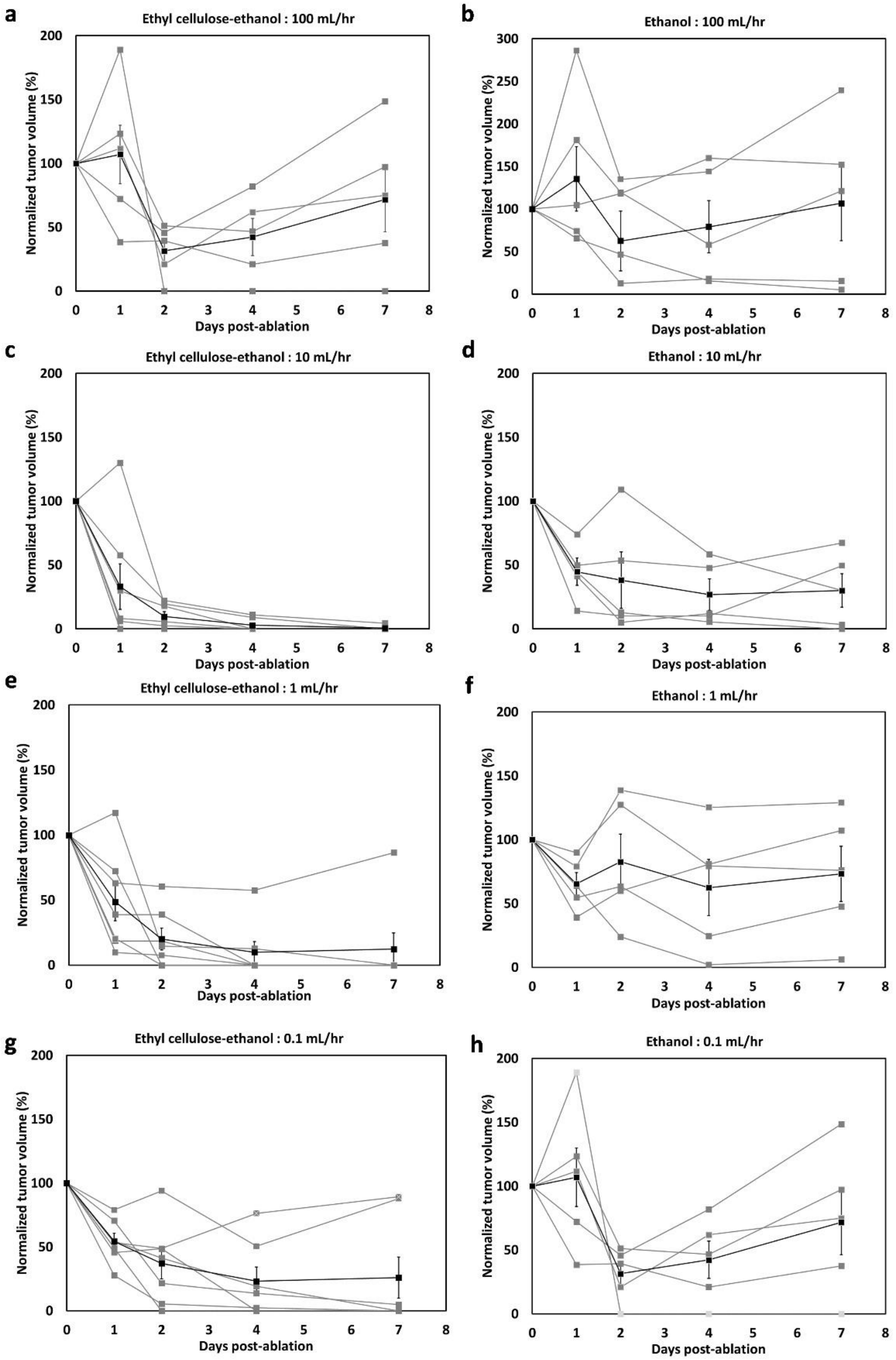

Supplementary Figure 2: Individual tumor responses for each injection rate and ethyl cellulose concentration combination are shown in gray with the average shown in black. Error bars depict standard error. All conditions had at least 5 samples and ethyl cellulose-ethanol injections at 0.1, 1 and 10 mL/hr had 7 samples.
